# Mutational analysis of the rotavirus NSP4 enterotoxic domain that binds to caveolin-1

**DOI:** 10.1186/1743-422X-10-336

**Published:** 2013-11-13

**Authors:** Judith M Ball, Megan E Schroeder, Cecelia V Williams, Friedhelm Schroeder, Rebecca D Parr

**Affiliations:** 1 Department of Pathobiology, Texas A&M University, TVMC, College Station, Texas 77843-4467, USA; 2 Present Address: Sandia National Laboratories, Albuquerque, New Mexico, USA; 3 Present Address: Texas State Veterinary Diagnostic Laboratory, College Station, TX 77843, USA; 4 Department of Physiology and Pharmacology, TAMU 4467, College Station, Texas 77843-4467, USA; 5 Present Address: Department of Biology, Stephen F. Austin State University, Nacogdoches, TX 75962, USA

## Abstract

**Background:**

Rotavirus (RV) nonstructural protein 4 (NSP4) is the first described viral enterotoxin, which induces early secretory diarrhea in neonatal rodents. Our previous data show a direct interaction between RV NSP4 and the structural protein of caveolae, caveolin-1 (cav-1), in yeast and mammalian cells. The binding site of cav-1 mapped to the NSP4 amphipathic helix, and led us to examine which helical face was responsible for the interaction.

**Methods:**

A panel of NSP4 mutants were prepared and tested for binding to cav-1 by yeast two hybrid and direct binding assays. The charged residues of the NSP4 amphipathic helix were changed to alanine (NSP4_46-175_-ala6); and three residues in the hydrophobic face were altered to charged amino acids (NSP4_46-175_-HydroMut). In total, twelve mutants of NSP4 were generated to define the cav-1 binding site. Synthetic peptides corresponding to the hydrophobic and charged faces of NSP4 were examined for structural changes by circular dichroism (CD) and diarrhea induction by a neonatal mouse study.

**Results:**

Mutations of the hydrophilic face (NSP4_46-175_-Ala6) bound cav-1 akin to wild type NSP4. In contrast, disruption of the hydrophobic face (NSP4_46-175_-HydroMut) failed to bind cav-1. These data suggest NSP4 and cav-1 associate via a hydrophobic interaction. Analyses of mutant synthetic peptides in which the hydrophobic residues in the enterotoxic domain of NSP4 were altered suggested a critical hydrophobic residue. Both NSP4_HydroMut112-140,_ that contains three charged amino acids (aa113, 124, 131) changed from the original hydrophobic residues and NSP4_AlaAcidic112-140_ that contained three alanine residues substituted for negatively charged (aa114, 125, 132) amino acids failed to induce diarrhea. Whereas peptides NSP4wild type _112__−140_ and NSP4_AlaBasic112-140_ that contained three alanine substituted for positively charged (aa115, 119, 133) amino acids, induced diarrhea.

**Conclusions:**

These data show that the cav-1 binding domain is within the hydrophobic face of the NSP4 amphipathic helix. The integrity of the helical structure is important for both cav-1 binding and diarrhea induction implying a connection between NSP4 functional and binding activities.

## Background

Rotaviruses (RV) induce a secretory and malabsorptive diarrhea, both of which are multi-factorial [1,2]. RV NSP4 is the first described viral enterotoxin and induces early secretory diarrhea in rodents [1,3-6]. Exogenously added NSP4 mobilizes intracellular calcium ([Ca^2+^]i) levels through an integrin receptor-mediated, phospholipase C (PLC)-dependent pathway [3,6], whereas endogenously expressed NSP4 mobilizes [Ca^2+^]i by a PLC-independent mechanism, i.e. by NSP4 functioning as a viroporin in the endoplasmic reticulum (ER) [7,8]. These data indicate unique activities of NSP4 in different environments. At the exofacial surface of the plasma membrane (PM), increasing evidence has established that NSP4 activates a calcium signal transduction pathway with the release of chloride and water into the lumen of the gut [9,10]. It has been hypothesized that *de novo* NSP4 is released from RV- infected cells whereupon it binds to neighboring or the same cell to initiate secretory diarrhea [5,11,12].

In addition to its enterotoxic activity, NSP4 performs multiple intracellular functions that contribute to RV morphogenesis and replication. Early reports show NSP4 is an ER transmembrane glycoprotein that serves as an intracellular receptor that binds double layered particles [13-16], and facilitates entry into the ER and acquisition of the outer coat to form triple layered particles with a transient ER membrane [10,15-17]. Subsequent silencing studies (siRNA) of RV-infected cells reveal that in the absence of NSP4 there are: (1) abnormal distributions of viral proteins in the viroplasm [18]; (2) little to no infectious viral particles present in the cell; (3) accumulations of empty viral particles [18,19], and (4) an up-regulation of viral transcription [19]. Taken together, NSP4 appears to function in viral pathogenesis, replication, and morphogenesis, as well as serving as an enterotoxin that induces calcium signaling events and fluid loss [20].

An NSP4 C-terminal cleavage fragment (residues 112-175) isolated from culture media of RV SA11-infected (MOI = 20; Sf9 cells) or NSP4 112-175-transfected cells show that at least a portion of NSP4 leaves the ER and the cell [21]. Bugarcic and Taylor report the secretion of a larger, alternately glycosylated 32 kD protein (MOI = 10, HT29 cells) [22], but signaling function of this larger NSP4 form was not reported. Other studies note the presence of NSP4 at multiple locations in RV SA11-infected cells [11,23-26]. For example, Boshuizen et al report the presence of NSP4 at the basolateral surface of polarized cells in association with the extracellular matrix proteins, laminin-beta 3 and fibronectin [11], while also showing evidence of apical release [22]. In addition, NSP4 colocalizes with LC3, an autophagic vesicle marker, tubulin, and VP5, a RV viroplasm marker [12,27,28]. The recent identification of integrins alpha 1 beta 1 and alpha 2 beta 1 as the NSP4 cell receptors support the previous observations that NSP4 binds to the outside of the cell and stimulates a PLC signaling pathway [12]. Our recent data show full-length (FL) NSP4 is released from intact cells and binds neighboring cells [29]. Taken together, these data indicate that a pool of NSP4 interacts with several host-cell molecules that may influence its movement from the ER to the outer leaflet of the PM and subsequent release from the cell.

Collectively, our previous data show (a) preferential binding of FLNSP4 and NSP4_114-135_ peptide to caveolae-like model membranes [30,31]; (b) transport of FLNSP4 to the PM and caveolae by a Golgi-bypassing, unconventional transport pathway [26]; (c) a direct interaction cbetween the structural protein of caveolae, caveolin-1 (cav-1), and NSP4 in yeast and mammalian cells [25]; (d) the presence of full length, endoglycosidase H (Endo-H)-sensitive FLNSP4 in caveolae microdomains isolated from PM fractions of infected cells [26]; (e) FLNSP4 traffics to the PM, exposing the C-terminus to the extracellular space and subsequently is released from the cell; and (f) K_d_ analyses of FLNSP4 and cav-1 peptides showed preferential binding to the N-terminus of cav-1, with an increased affinity in the presence of cholesterol-rich model membranes, as well as a direct interaction between FLNSP4 and cholesterol [32].

Cav-1 is localized in the intestine, interacts with cholesterol, functions to transport de novo synthesized cholesterol to and from the ER and caveolae, and organizes signaling molecules, including those regulating calcium homeostasis [33-35]. The NSP4 binding site for cav-1 maps to aa 114-135, the enterotoxic domain, using *in vivo* yeast two-hybrid (Y2H) assays, *in vitro* peptide binding assays, co-immunoprecipitation (co-IP) reactions, and laser scanning confocal microscopy (LSCM) co-localization and fluorescent resonance energy transfer (FRET) analyses [25]. The enterotoxic domain of NSP4 interacts with both the N- and C-termini of cav-1 [36]. This result was surprising as cav-1 forms a hairpin loop in the cytofacial leaflet of the PM such that the two termini appear physically separated in the cytosol of the cell [37-41].

The goal of this study was to resolve the details of the NSP4-cav-1 interaction by (i) elucidating the type of interaction between NSP4 and cav-1, (ii) mapping the precise binding residues, and (iii) determining the extent to which interaction with cav-1 influences enterotoxic activity.

## Results

### The hydrophobic face of the NSP4 amphipathic alpha helix (AAH) binds cav-1

**Figure 1:**
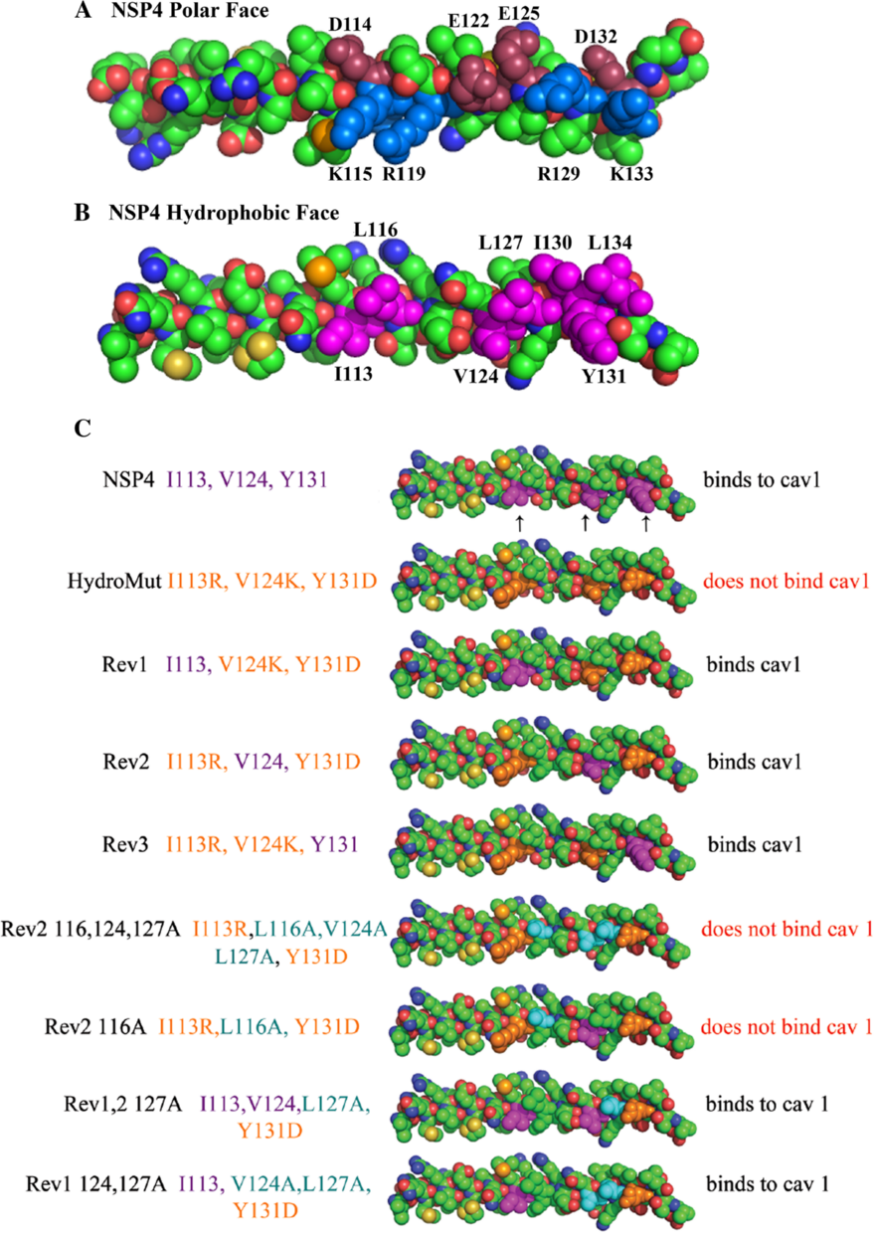
**A and B Organization of the Polar and Hydrophobic faces of the NSP4 crystal structure of the enterotoxic AAH (A and B respectively). A** shows the acidic amino acids, D114, E122, E125 and D132 depicted in a maroon color. The basic amino acids K115, R119, R129 and K133 are shown in blue. **B** shows the hydrophobic amino acids of the AAH of NSP4 I113, L116, V124, L127, I130, Y131 and L134 in purple. **C** PyMol representations of the hydrophobic face of NSP4-(residues 46-175) and mutants. Amino acids in purple (I113, V124 and Y131) are indicated by arrows represent the wild type NSP4. Eight mutant clones are viewed below the wild type NSP4. The mutations, I113R, V124D and Y131K are orange, and amino acids that are mutated to alanines are shown as blue. To the right of each represented clone, the results of the yeast two hybrid and the peptide binding assays are given.

To identify the face of the amphipathic helix that binds cav-1, a 3-D structural model was generated using the crystallographic determinants for NSP4 95-135 [42] and visualized using PyMol (The PyMOL Molecular Graphics System, Version 1.2r3pre, Schrödinger, LLC). The six charged residues between 114 and 135 were mutated to alanine (D114A, K115A, R119A, E125A, D132A, and K133A) in FLNSP4-Ala6, and three hydrophobic residues were mutated to charged residues (I113R, V124K, and Y131D), in FLNSP4-HydroMut (Figure 1A and B, Table 1).

**Table 1:** NSP4 constructs and primers

**Name of construct**^**a**^	**Amino acid changes**^**b**^	**Primers for site directed mutagenesis**^**c**^
FLNSP4 Ala (1-175)	D114A,K115A,R119A	BP3 5′ TACCGCGGCCGCGGAAAAGCTTACCGACCTCA 3′
	E124A,D132A,K133A	BP28 5′ TACTTGTTCAATTTCAGCTGTAGTCAATGCGGCAATCATTT 3′
		BP29 5′AAATTGAACAAGTAGCTTTGCTTAAACGCATTTACGCTGCATTGA 3′
		BP4 5′ TCTAGATATCTCGAGTTACATTGCTGCAGTCACTT 3′
NSP4 Ala (46-175)	D114A,K115A,R119A	BP23 5′ ATTTAACCATGGCACTACATAAAGCATCCATTCCA 3′
	E124A,D132A,K133A	BP4 5′ TCTAGATATCTCGAGTTACATTGCTGCAGTCACTT 3′
FLNSP4 HydroMut (1-175)	I113R,V124K,Y131D	BP3 5′ TACCGCGGCCGCGGAAAAGCTTACCGACCTCA 3′
		BP31 5′ TTGTTCAATTTCACGTGTAGTCAATTTGTCACGCATTTCT 3′
		BP30 5′ TGAAATTGAACAAAAAGAGTTGCTTAAACGCATTGACGATAA 3′
		BP4 5′ TCTAGATATCTCGAGTTACATTGCTGCAGTCACTT 3′
NSP4 HydroMut (46-175)	I113R,V124K,Y131D	BP23 5′ ATTTAACCATGGCACTACATAAAGCATCCATTCCA 3′
		BP4 5′ TCTAGATATCTCGAGTTACATTGCTGCAGTCACTT 3′
Rev1 (46-175)	I113,V124K,Y131D	BP23 5′ ATTTAACCATGGCACTACATAAAGCATCCATTCCA 3′
		BP34 5′ TTGTTCAATTTCACGTGTAGTCAATTTGTCAATCATTTCT 3′
		BP30 5′ TGAAATTGAACAAAAAGAGTTGCTTAAACGCATTGACGATAA 3′
		BP4 5′ TCTAGATATCTCGAGTTACATTGCTGCAGTCACTT 3′
Rev2 (46-175)	I113R,V124,Y131D	BP23 5′ ATTTAACCATGGCACTACATAAAGCATCCATTCCA 3′
		BP31 5′ TTGTTCAATTTCACGTGTAGTCAATTTGTCACGCATTTCT 3′
		BP32 5′ TGAAATTGAACAAGTAGAGTTGCTTAAACGCATTGACGATAA 3′
		BP4 5′ TCTAGATATCTCGAGTTACATTGCTGCAGTCACTT 3′
Rev3 (46-175)	I113R,V124K,Y131	BP23 5′ ATTTAACCATGGCACTACATAAAGCATCCATTCCA 3′
		BP31 5′ TTGTTCAATTTCACGTGTAGTCAATTTGTCACGCATTTCT 3′
		BP33 5′ TGAAATTGAACAAAAAGAGTTGCTTAAACGCATTTACGATAA 3′
		BP4 5′ TCTAGATATCTCGAGTTACATTGCTGCAGTCACTT 3′
Rev2 116,124,127A	I113R,L116A,V124A,L127A,Y131D	BP23 5′ ATTTAACCATGGCACTACATAAAGCATCCATTCCA 3′
		BP80 5′ TTCAATTTCACGTGTAGTTGCTTTGTCTGC 3′
		BP81 5′ AAATTGAACAAGCAGAGTTGGCAAAACGCGCAGA 3′
		BP4′ 5′ TTACATTGCTGCAGTCACTTCTCTTGGTT 3′
Rev2 116A	I113R,V124,L116A,Y131D	BP23 5′ ATTTAACCATGGCACTACATAAAGCATCCATTCCA 3′
		BP91 5′ TTGTTCAATTTCACGTGTAGTTGCTTTGTCAATCATTTCT 3′
		BP32 5′ TGAAATTGAACAAGTAGAGTTGCTTAAACGCATTGACGATAA 3′
		BP4′ 5′ TTACATTGCTGCAGTCACTTCTCTTGGTT 3′
Rev1,2 127A	I113,V124,L127A,Y131D	BP23 5′ ATTTAACCATGGCACTACATAAAGCATCCATTCCA 3′
		BP89 5′ AATGCGTTTTGCCAACTCTT 3′
		BP88 5′ AAGTGGAGTTGGCAAAACG 3′
		BP4′ 5′ TTACATTGCTGCAGTCACTTCTCTTGGTT 3′
Rev1 124,127A	I113,V124A,L127A,Y131D	BP23 5′ ATTTAACCATGGCACTACATAAAGCATCCATTCCA 3′
		BP91 5′ TTGTTCAATTTCACGTGTAGTTGCTTTGTCAATCATTTCT 3′
		BP81 5′ AAATTGAACAAGCAGAGTTGGCAAAACGCGCAGA 3′
		BP4′ 5′ TTACATTGCTGCAGTCACTTCTCTTGGTT 3′

^a^Names of mutants of full length NSP4 or NSP4 46-175 that were cloned into the yeast two hybrid vectors, pDest32 and pDest22 and the yeast expression vector, pYDest52.

^b^Native amino acids of NSP4 46-175 that were mutated or reverted back to the original amino acids.

^c^The names and the sequences of the oligonucleotides that were used to clone the mutant clones.

The interactions of these FLNSP4 mutants with cav-1 were evaluated by yeast-two-hybrid (Y2H) assays by reacting the Gal4 activation domain fusion protein with the NSP4:Gal4 binding domain fusion proteins. The plasmids were co-transformed into the yeast strain MaV203 as previously described [25]. The growth patterns of the co-transformed yeast confirmed the activation of the three reporter genes of the Y2H assay and established FLNSP4-Ala6 as positive for interacting with cav-1. In contrast, the FLNSP4-HydroMut failed to activate the three reporter genes indicating a lack of binding (data not shown).

Previous Y2H assays show that the reactions are clearer in the absence of the first two N-terminal hydrophobic domains of FLNSP4 [25]. Therefore, new mutants, NSP4, NSP4_Ala6_, and–_HydroMut_, that lack the first two hydrophobic domains, were cloned into the Gateway® entry clone, pENTR11, and inserted into the pDest32 plasmids to produces the Gal4 binding domain fusion proteins. The Gateway® entry clone, pECav-1 was used to insert the cav-1 sequence into the pDest22 plasmid that produces a fusion protein with the Gal4 activating domain for Y2H assays [25].

The growth patterns of the co-transformed yeast were monitored for activation of transcription from three chromosomally integrated reporter genes *Ura3, His3,* and *lacZ* (Table 2). To assess the success of the co-transformation and transcription activation, four growth phenotypes were determined using media lacking the following amino acids: 1) Leucine and tryptophan; 2) Leucine, tryptophan, and histidine with 50 mM 3-Amino-1,2,4-Triazol (3-AT); 3) Leucine, tryptophan, and uracil; 4) Leucine, tryptophan, with 5-fluoroorotic acid (5FOA). The growth patterns were qualitatively scored and compared to the phenotypes that are defined in the Y2H manual [43] as positive and negative interactions (data not shown).

**Table 2:** **Phenotypes of pD32-NSP4 mutants plus pD22-caveolin-1**^**a**^

	**CSM Leu**^**-**^**Trp**^**-**^**His**^**-**^ **+ 3AT**	**CSM Leu**^**-**^**Trp**^**-**^**Ura**^**-**^	**CSM Leu**^**-**^**Trp**^**-**^ **+ 0.2% 5FOA**	**beta-gal**	**Phenotypes**^**c**^
	**50 mM**			**Units**^**b**^	
Negative Control	+/−	-	-	0.134	**Negative**
NSP4 46-175	+/−	-	+/−	1.390	Positive
NSP4 Ala	-	-	+/−	1.822	Positive
NSP4 HydroMut	-	+/−	+	0.105	**Negative**
Rev1	-	-	+/−	3.941	Positive
Rev2	+/−	-	+/−	3.042	Positive
Rev3	+/−	+/−	-	3.195	Positive
Rev1,2 127A	+/−	-	+/−	2.581	Positive
Rev1 124,127A	-	-	+/−	4.044	Positive
Rev2 116A	-	-	+/−	0.165	**Negative**
Rev2 116,124,127A	+/−	+/−	+/−	0.056	**Negative**

^a^Colonies were grown on CSM Leu^-^Trp^-^ and replica plated onto CSM Leu^-^Trp^-^His with 50 mM 3AT; CSM Leu^-^Trp^-^Ura^-^; and CSM Leu^-^Trp^-^ with 0.2% 5FOA.

^b^Beta-galactosidase activity was quantitatively measured by CPRG assays.

^c^The phenotype was determined by combining the results of growth on differential media and beta-galactosidase activity.

The negative control and mutants NSP4 clones that did not interact with caveolin-1 are in bold letters.

Induction of the *lacZ* reporter gene was confirmed using the quantitative chlorophenol red-beta-D-galactopyranoside (CPRG) assay for beta-galactosidase activity and reported as beta-galactosidase units (BGU). The negative yeast control had 0.134 BGU while truncated NSP4_46-175_ had 1.39 BGU. NSP4_HydroMut_ and NSP4_Ala6_ had 0.105 and 1.822 BGU, respectively, indicating a negative and positive interaction, respectively (Table 2).

The growth patterns of the co-transformed yeast confirmed the activation of the three reporter genes of the Y2H assay. The N-terminally deleted NSP4_Ala6_ showed a positive reaction for interacting with cav-1. In contrast, the N-terminally deleted NSP4_HydroMut_ failed to activate the three reporter genes. These data suggested alteration of the hydrophobic face of the amphipathic alpha helix of the NSP4 enterotoxic domain (I113, V124, and Y131), failed to bind cav-1.

### NSP4_HydroMut_ failed to associate with cav-1 by a peptide binding/pull-down assay

**Figure 2:**
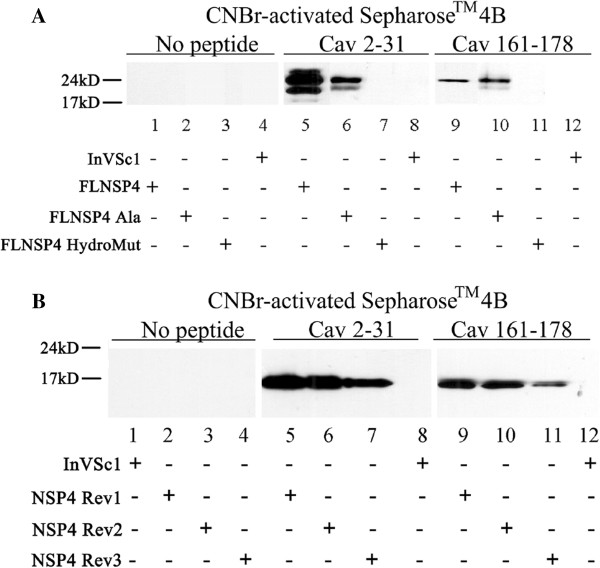
**Binding of Caveolin-1 Peptides to NSP4 Constructs.** Panel **A**: Transduced yeast lysates expressing full-length- NSP4,-NSP4-Ala6 and -NSP4-HydroMut (FLNSP4, FLNSP4-Ala6, and FLNSP4-HydroMut) were incubated with CNBr-activated sepharose 4B beads bound by the N-terminal (cav-1_2-31_), C-terminal (cav-1_161-178_), or beads only. The beads were washed and the captured proteins separated by SDS-PAGE, transferred to nitrocellulose, and probed using rabbit anti-NSP4_150-175_. Controls (lanes 1-4) show the absence of non-specific binding to the sepharose beads with all lysates tested. Lanes 5-7 show the reactivity of FLNSP4 and-NSP4 mutants with cav-1_2-31_. Only the FLNSP4-HydroMut failed to bind the N-terminal caveolin-1 peptide. The same binding pattern was observed when FLNSP4 and -NSP4 mutant proteins where incubated with cav-1_161-178_ (lanes 9, 10, and 11). InVSc1 alone showed no NSP4-specific bands using either peptide (lanes 8 and 12). Panel **B**: Western blot analyses of CNBr-activated sepharose 4B beads that were bound by cav-1_2-31_, cav_161-178_ or no peptide and reacted with yeast lysates expressing Rev113I, Rev124V, Rev131Y. The peptide-bound proteins were detected by Western blot using rabbit anti-NSP4_150-175_. Lanes 1-4 demonstrate that the NSP4 proteins failed to bind the sepharose beads alone with all lysates tested. Lanes 5, 6, and 7 indicate that all three revertant NSP4 proteins bound to the N-terminal peptide, Cav-1_2-31_. The same binding pattern was observed with the C-terminal peptide, cav-1_161-178_ (lanes 9, 10, and 11), while InVSc1 failed to bind either peptide (lanes 8 and 12).

To confirm the Y2H results, newly synthesized cav-1 peptides (amino acids 2-31, 76-101, and 161-178) were bound to sepharose beads, and reacted with yeast lysates expressing FLNSP4, FLNSP4-Ala6_,_ or FLNSP4-HydroMut (Figure 2A). As expected, Western blot analyses of precipitated lysates with sepharose beads only and precipitated untransformed lysates did not demonstrate NSP4-specific bands (Figure 2A, lanes 1-4, 8, and 12). In FLNSP4 containing lysates, NSP4 was pulled down by cav_2-31_ and showed NSP4-specific bands at 28, 26 and 20 kD that correspond to double, single and non-glycosylated forms of NSP4, respectively (Figure 2A, lane 5). FLNSP4-Ala6 also bound to cav_2-31_ but exhibited only two bands at ~26 and 20 kD (Figure 2A, lane 6). The double glycosylated form was not detected under these conditions. A 26 kD, NSP4-specific band similarly was visible with both FLNSP4 and FLNSP4-Ala6 when captured with cav_161-178_ (Figure 2A, lanes 9 and 10). In agreement with the Y2H data, FLNSP4-HydroMut lysates failed to react with either of the sepharose-bound peptides, cav-1_2-31_ or cav-1_161-178_ (Figure 2A, lanes 7 and 11). These results support the hypothesis that the binding of NSP4 to cav-1 is via a hydrophobic interaction.

To illustrate FLNSP4 and NSP4_46-175_ yield similar results, the same cav-1 peptides were tested with NSP4_46-175_, NSP4_Ala6_ and NSP4_HydroMut_ and yielded identical results as that acquired with FLNSP4, FLNSP4-Ala6, and FLNSP4-HydroMut (data not shown). The three hydrophobic amino acids changed in NSP4_HydroMut_, I113R, V124K, and Y131D, appeared to be important for the binding to cav-1. Peptides corresponding to both the N- and C-terminal regions of cav-1 (2-31, 19-40 and 161-178) interacted with NSP4 but cav-1 residues 76-101 failed to bind NSP4 (data not shown) as previously reported [36].

### All three NSP4_46-175_HydroMut revertants associate with cav-1 by Y2H analyses and peptide pull-down assays

To further localize the cav-1 binding domain of the N-terminal deletion mutant (NSP4_46-175)_, each of the mutant residues in NSP4_HydroMut_ individually were reverted back to the original hydrophobic amino acid [Rev1(113I), Rev2(124 V) and Rev3(131Y), Table 1], and transformed into yeast with cav-1 for evaluation by Y2H assays. All three revertants showed growth patterns of the co-transformed yeast confirming the activation of the three reporter genes of the Y2H assay (data not shown). Induction of the *lacZ* reporter gene was confirmed using the quantitative CPRG assay for beta-galactosidase activity reported as BGU. The revertants (Rev1, Rev2, Rev3) yielded 3.941, 3.042, and 3.195 BGU, respectively, which was 30 times higher than the negative yeast control (0.134 BGU), and over 2 times higher than NSP4_46-175_ (1.39 BGU) (Table 2). These data indicated all 3 revertants interacted with cav-1, so a single hydrophobic residue between NSP4 124-131 was sufficient for binding.

To validate the Y2H data, cav-1 peptides (aa 2-31, 76-101, and 161-176) were bound to sepharose beads and tested for capturing each of the individual revertants (Figure 2B). Western blot analyses of Rev1, Rev2, and Rev3 when reacted with sepharose beads only or untransformed yeast lysates (InVSc1) showed no bands (Figure 2B, lanes 1-4, 8 and 12). An NSP4-specific band was observed at 17 kD with all three revertants when reacted with both cav_2-31_ and _161-178_ (lanes 5, 6, 7 and 9, 10, 11 respectively), which is the correct theoretical molecular weight of NSP4 46-175. NSP4-specific bands were absent when the lysates were incubated with cav_76-101_ (data not shown). These data support the *in vivo* Y2H results showing cav-1 interacted with each of the revertants.

### NSP4- and cav-1-fusion proteins are present in the co-transformed yeast

**Figure 3:**
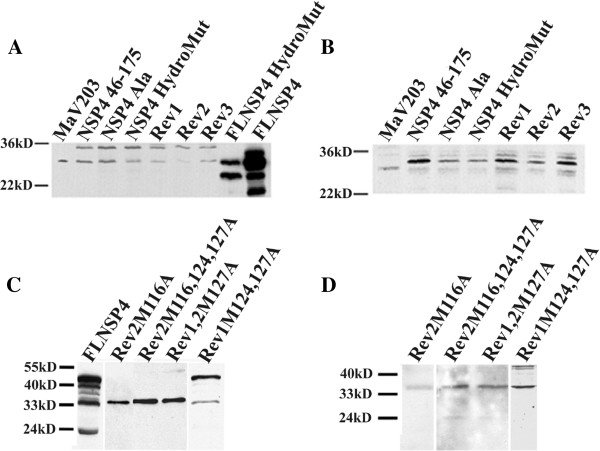
**Representative Western blot analyses of yeast lysates co-transformed with NSP4 or NSP4 mutants with caveolin-1 fusion proteins.** The presence of NSP4- and cav-1 fusion proteins in the Y2H assay was shown by Western blot analyses of the yeast co-transformed with pD22cav-1 and each of the following fusion proteins: pD32NSP4 46-175 (lanes 2A and 2B), pD32NSP4-Ala6 (lanes 3A and 3B) , pD32NSP4-HydroMut (lane 4), pD32Rev113I (lane 5), pD32Rev124V (lane 6), and pD32Rev131Y (lane 7). Lanes 8 and 9 show the FLNSP4-HydroMut and FLNSP4, respectively. Blots were probed using **(A)** rabbit anti-NSP4_150-175_ or **(B)** rabbit anti-cav-1_2-31_. Panel **A**: The non-transformed yeast (MaV203, lane 1) showed a non-specific band at ~29 kD that was observed in all lanes. Lysates expressing FLNSP4-HydroMut (lane 8) demonstrated NSP4-specific bands at ~ 28, 24 kD. Lysates expressing FLNSP4 showed bands at ~ 28, 24, and 20 kD (lane 9). All of the co-transformed yeast demonstrated a NSP4-fusion protein band at ~ 34.4 kD (**A**, lanes 2-7). Panel **B** revealed a faint contaminating band below the 34.9 kD specific caveolin-1-fusion protein band (lanes 1-7). Lanes 2-7 show the specific caveolin-1 fusion protein band at ~34.9 kD. Panel **C**: Lysates expressing FLNSP4 (lane 1) showed bands at ~ 24 and 28 kD and (lane 9). All of the co-transformed yeast demonstrated both monomeric and multimeric NSP4-specific fusion protein bands (lanes 1). Rev2M116A, Rev2M116,124,127A, Rev1,2M127A and Rev1M124,127A revealed the ~34.9 kD specific NSP4-1-fusion protein band (lanes 2-5). Also, Rev1M124,127A demonstrated a multimeric NSP4-fusion specific band similar to one observed with FLNSP4. Panel **D**: Rev2M116A, Rev2M116,124,127A, Rev1,2M127A and Rev1M124,127A revealed a ~ 34.9 kD specific caveolin-1-fusion protein band (lanes 1-4). Also, Rev1M124,127A demonstrated a multimeric caveolin-1-fusion protein band.

To verify the presence of the NSP4 and cav-1 GAL-4 fusion proteins in the yeast that showed no binding to cav-1 in the Y2H assay, the co-transformed lysates were probed with either anti-NSP4_150-175_ (Figure 3A and C) or anti-cav-1_2-31_ (Figure 3B and D) peptide-specific antibodies. Controls included untransformed MaV203 yeast lysates. The controls failed to show an antibody-specific band at the correct molecular weight of the fusion proteins for both the activating domain (AD)-NSP4 and binding domain (BD)-cav-1. However, a non-specific band at ~28 kD was observed when blotted with both antisera (Figure 3, lane 1). All NSP4 and cav-1 fusion proteins tested expressed at the correct molecular weight (34.4 kD and 34.9 kD, respectively) and were reactive with NSP4- (Figure 3, panels A&C) and cav-1 peptide-specific antisera (panel B&D). These data established that the encoded sequences from both plasmids were translated as fusion proteins in the co-transformed yeast, even when no reactivity was detected.

### Four mutations of the revertants demonstrate a key hydrophobic residue in binding cav-1

Four additional mutants were generated from the revertants, Rev2 116,124,127A; Rev2 116A; Rev1,2 127A; and Rev1 124 ,127A (Tables 1 and 2, Figure 1C), individually were co-transformed with cav-1 and were evaluated by Y2H analyses. Rev1,2 127A and Rev1 124,127A revealed positive patterns of growth for a protein:protein interaction while Rev2 116,124,127A and Rev2 116A demonstrated negative growth patterns (data not shown). All four revertant mutant clones quantitatively were tested for beta-galactosidase activity. Rev2 116,124,127A and Rev2 116A both demonstrated beta-gal activity of 0.056 and 0.165 BGU, respectively, which was less than or equivalent to values of the negative control (0.134 BGU) (Table 2). In contrast, Rev1 124,127A and Rev1,2 127A displayed β-gal activity of 4.044 and 2.581 BGU respectively, which was approximately 20-30 times higher than that of the negative control (0.134 BGU) (Table 2). These data signify that Rev1 124,127A and Rev1,2 127A bound cav-1, whereas Rev2 116,124,127A and Rev2 116A lacked binding to cav-1.

### *In vitro* cav-1 peptide binding assays of the revertants confirm the Y2H results

**Figure 4:**
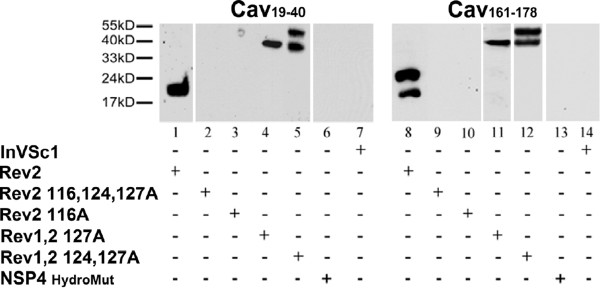
**Western blot analyses of caveolin.** Yeast lysates expressing Rev2; Rev2 116, 124,127A; Rev2 116A; Rev1,2 127A; Rev1,2 124,127A, and NSP4_HydroMut_ were incubated with CNBr-activated sepharose 4B beads bound by either the N-terminal (cav-1_19-40_) (lanes 1-7) or C-terminal (cav-1_161-178_) (lanes 8-14) peptides. Left Panel: Yeast lysates were reacted with bound cav-1 residues 19-40 and probed with rabbit anti-NSP4_150-175_. Lanes 1, 4, and 5 depict the reactivity of Rev2; Rev1,2 127A; and Rev1,2 124,127A, respectively. Lanes 2, 3, 6, and 7 show a lack of reactivity with Rev2 116, 124,127A; Rev2 116A; NSP4_HydroMut_; and InVSc1 respectively. Right Panel: The same binding pattern was observed when the yeast lysates were incubated with cav-1_161-178_ (lanes 8-14). RV-infected MDCK cell lysates (lane 15) demonstrate the specificity of the rabbit anti-NSP4_150-175_. Non-specific binding to the beads alone was not observed (data not shown). Lanes 4, 5, 11, and 12 show multimeric forms of NSP4.

Previously, we had reported that cav_19-40_ binds to NSP4 with a higher affinity than cav_2-20_ and cav_161-178_[32]. Therefore, we employed the cav_19-40_ peptide for the remainder of the binding studies. Cav_19-40_ and cav_161-178_ were bound to sepharose beads, reacted with the revertant mutants expressed in yeast, and assessed by Western blot. Lysates from untransformed InVSc1 yeast (Figure 4, lanes 7 and 14) and all NSP4 mutant proteins incubated with only sepharose beads demonstrated no reactivity to NSP4 by Western blot (data not shown). Further, no NSP4 specific bands were detected by Western blot of lysates expressing Rev2 116,124,127A, Rev2 116A or NSP4_HydroMut_ incubated with cav_19-40_ or cav_161-178_ (Figure 4, lanes 2, 3, 6; lanes 9, 10, 13). Only Rev1,2 127A, Rev1 124,127A, and RV-infected cell lysates showed binding to cav-1 residues 19-40 and 161-178 (Figure 4, lanes 1, 4, 5, 8, 11 and 12). Rev 2 bound cav_19-40_ and _161-178_ as a monomer (~17 Kd, Figure 4 lanes 1 and 8). With cav_161-178_, a NSP4 specific band at ~21 Kd (lane 8), also was observed. Two rev2 mutants likewise bound both cav-1 peptides (lanes 4, 5 and 11, 12), but only bound the NSP4dimeric form. A larger dimer also was present in lanes 5 and 12, further indicating the larger molecular weight band is a multimeric form of NSP4. Together, these data revealed that L116 is important for a protein-protein interaction with cav-1.

### Mutations within the amphipathic helix of NSP4 peptides abolish diarrheagenic activity in mouse pups

Our previous study delineates the cav-1 binding site between NSP4 residues 114-135 (24). Hence, three peptides encompassing amino acids (aa) 112-140 of NSP4 were synthesized, which contained mutated residues in the hydrophobic (NSP4_Hydro112-140_), acidic (NSP4_AlaAcidic112-140_) or basic (NSP4_AlaBasic112-140_) face of the amphipathic alpha-helix (AAH). The sequences of each of the NSP4 mutant peptides are given in Additional file 1: Table S1. NSP4_Hydro112-140_ contains three charged amino acids (aa113, 124, 131) that were altered from the original hydrophobic residues. NSP4_AlaAcidic112-140_ and NSP4_AlaBasic112-140_ each contained three alanine residues substituted for negatively charged (aa114, 125, 132) and positively charged (aa115, 119, 133) residues, respectively.

Balb/C mouse pups (6-10 days) were administered wild type (wt) or mutant peptides by the intraperitoneal route and monitored for diarrhea as previously described [3]. Prior to injection, all purified peptide samples were tested for endotoxin by the *Limulus* Amebocyte Lysate (LAL) test (Associates of Cape Cod, Inc.) to ensure that samples were endotoxin free. A value of 0.5 EU/mL or less was considered acceptable for use in the diarrhea studies. Mouse pups injected with endotoxin free PBS served as a negative control. Mouse pups were monitored for diarrhea every 2 hours for 12 hours and then at 24 hours post injection. The severity of the diarrhea was scored on a scale of 1 to 4 (data not shown) (5). Following injection, both the wt-NSP4_112-140_ and NSP4_AlaAcidic112-140_ peptides caused diarrhea in 50% and 67% of the mouse pups tested, respectively (Additional file 1: Table S1). NSP4_Hydro112-140_ and NSP4_AlaBasic112-140_ peptides, however, did not induce diarrhea in any of the mouse pups tested. These preliminary results suggest that residues within both the hydrophobic face and the basic face of the amphipathic alpha-helix of NSP4 are also important for enterotoxic function.

### The mutant NSP4 peptides had an altered secondary structure when compared to NSP4 _112-140wt_

**Figure 5:**
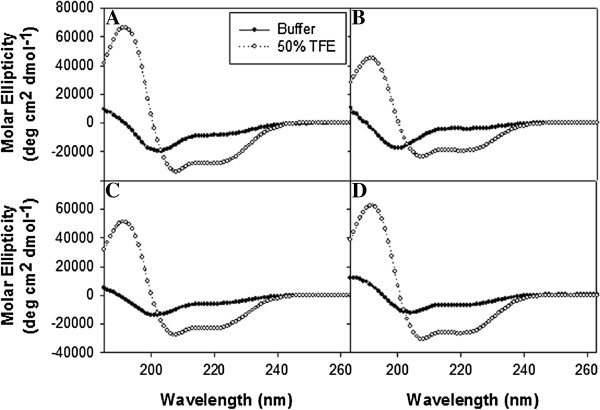
**CD spectra of NSP4 peptides in aqueous buffer (**●**) and 50**% **TFE (**○**). (A)** wtNSP4_112-140_; **(B)** NSP4_HydroMut112-140_; **(C)** NSP4_AlaAcidic112-140_; **(D)** NSP4_AlaBasic112-140_. X axis represents the wavelength (nm) and the y axis is the Molar Ellipticity (deg cm^2^ dmol^-1^).

To determine any alteration in structure of the mutant peptides, which may influence function, circular dichroism (CD) was employed. CD spectra of the mutant NSP4 (mtNSP4) peptides in aqueous buffer differed from that of the wild-type peptide (Figure 5B-D, dark circles). The alpha-helical content was calculated based on the molar ellipticity values at 222 nm. The NSP4_HydroMut112-140_ peptide seemed most affected by the mutations made in the AAH, as the alpha-helical content was determined to be 19.4 ± 2.2% (Additional file 1: Table S1). The mutations made in each of the charged faces of the AAH had less of an effect on alpha-helix formation within the corresponding peptides. The alpha-helical content for the NSP4_AlaAcidic112-140_ and NSP4_AlaBasic112-140_ peptides was determined to be 23.7 ± 0.6% and 26.2 ± 2.9%, respectively (Additional file 1: Table S1).

In the presence of 50% trifluoroethanol (TFE, known to promote a hydrophobic environment and to enhance folding of synthetic peptides) each of the mutant NSP4 peptides increased in their alpha-helical secondary structure (2.5-3 times that in aqueous buffer) (Figure 5B-D, open circles). As in aqueous buffer, the NSP4_HydroMut112-140_ peptide exhibited less alpha-helical formation (57.8 ± 0.6%) than the NSP4_AlaAcidic112-140_ and NSP4_AlaBasic112-140_ peptides (73.1 ± 7.3% and 75.3 ± 2.8%, respectively) Additional file 1: Table S1. Therefore, the mutations made within the hydrophobic face of the AAH/cav-1 binding domain of NSP4 most negatively affected the alpha-helix folding of the peptide. Mutation of residues within the charged faces, however, only had a slight affect (NSP4_AlaAcidic112-140_) or no affect (NSP4_AlaBasic112-140_) on the alpha-helix structure of the peptides.

### Conservation of key NSP4 residues between different RV strains

**Figure 6:**
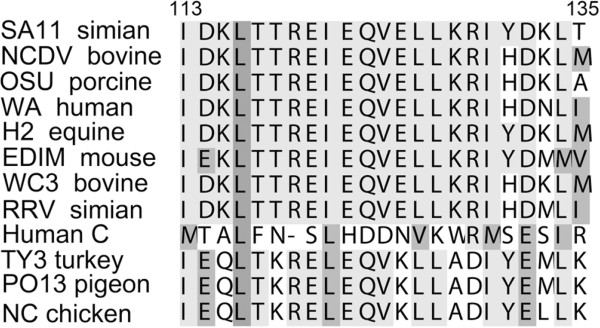
**Rotavirus NSP4 protein alignments.** NSP4 protein sequences obtained from GenBank of twelve rotavirus strains that represent groups A through E. [GenBank: ABZ04174, BAA24144, ABZ04170, BAB83830, AAA64924, AAL11029, AAB58698, AAD50676, ABV66094, BAA13728, P08434, AND P04512] were aligned with CLUSTALW for multiple sequence alignments [51,52].

NSP4 amino acids 113-135 obtained from GenBank of twelve RV strains that represent groups A through E. (accession no. **ABZ04174, BAA24144, ABZ04170, BAB83830, AAA64924, AAL11029, AAB58698, AAD50676, ABV66094, BAA13728, P08434, AND P04512**) were aligned with CLUSTAL (Figure 6). The following observations were made: (i) all aligned sequences included I113 and L127 except Human RV C I113M and L127K; (ii) all twelve strains contained L116; (iii) position 124 was V with one exception, Human RV C V124D; (iv) L134 was conserved with the exception of EDIM L134M and Human RV C L134I, another hydrophobic amino acid; and the residue 131 varied from Y131, H131 (NCDV, OSU, Wa, RRV), S131 (Human RV C). The high similarity of I113, L116, V124, L127, and L134 infer the importance of these residues. Given the high sequence divergence recently reported in NSP4 sequences, the conservation of the hydrophobic residues is all the more remarkable [44].

## Methods

### Antibodies

NSP4 and cav-1 peptide-specific antibodies were generated in New Zealand white rabbits to NSP4 residues 150-175 and cav-1 residues 2-31, respectively, following established protocols (5). Antibodies were purified against the peptide and used as primary antibodies in Western blots as previously described [25]. All primary antibodies were detected by horseradish peroxidase (HRPO)-labeled goat anti-rabbit IgG (Pierce, Rockford, Il).

### Mammalian cell lines and yeast strains

MDCK cells were obtained from ATCC (Rockville, MD) and maintained in Dulbecco modified Eagle medium (DMEM; Gibco, Grand Island, NY) supplemented with 10% fetal bovine serum (FBS), glutamine (2 mM), penicillin-streptomycin (100 ug/ml) and non-essential aa (1×) (Sigma, St. Louis, MO). To compare the NSP4 expressed in yeast to that expressed in RV-infected mammalian cells, MDCK cells were infected with RV SA114F, lysates were collected, and subjected to Endo H digestion as previously described [45,46]. The cell lysates also were utilized as a positive control with the pulldown assays.

*Saccharomyces cerevisiae* strain MaV203 (*MAT*α, *leu*2-3,112, *trp*1-901, *his*3Δ200, *ade*2-101, *gal*4Δ, *gal*80Δ, *SPAL*10::*URA*3, *GAL1*::*lacZ*, *HIS*3_uas gal1_::*HIS*3@*LYS*2, can1^r^, cyh2^r^) was used for all Y2H analyses [43,45,46]. A collection of yeast strains that contain plasmid pairs expressing fusion proteins with a spectrum of interaction strengths [pPC97 (*GAL4*-DB, *LEU2*), pPC97-*CYH2*^S^ and pPC86 (*GAL4*-AD, *TRP1*)] were used as controls [43,47]. The control plasmids pDBleu and pEXP-AD507 contain only the *Gal4* DNA-binding domain (BD) and the *Gal4* activating-domain (AD) respectively. The *S. cerevisiae* yeast strain InVSc1 (MATα *his3-*Δ*1*, *leu2*, *trp1-289*, *ura3-52*; Invitrogen) was used to express native and mutant NSP4 proteins that were used in peptide binding studies.

### NSP4 cloning strategy

PCR products of NSP4 (residues 46-175), NSP4_Ala6_ and NSP4_HydroMut_ constructs were directionally cloned into the Invitrogen Gateway™ Entry vector (pENTR11, Invitrogen, CA), sequence verified, and cloned into the Invitrogen Gateway™ destination vectors, pDEST22 and pDEST32, as previously described [25,43,48]. All plasmid manipulations were performed according to standard protocols in the *Escherichia coli* strains DH5α as described in the Gateway™ System manual [49].

To further refine the specific NSP4-cav-1 binding site, seven NSP4_46-175_ mutants were constructed and sequence verified (Rev113I, Rev124V, Rev131Y, Rev116,124,127A, Rev124V,116A, Rev113I,124 V,127A and Rev113I,124 V,127A, Table 1 and Figure 1C). The three amino acids mutated in NSP4_HydroMut_ (I113R, V124K and Y131D) were individually reverted back to the original amino acid (Rev1-I113; Rev2-V124; and Rev3-Y131; Figure 1C). The next four mutants were derived from combinations of alanine substitutions and reverting charge amino acid to the original hydrophobic amino acids in Rev2. Alanines were used to minimize conformational and structural disruption of the hydrophobic face of the AAH.

### Yeast two-hybrid screening

The MaV203 yeast strain was co-transformed with cav-1 and one of the NSP4 constructs (FLNSP4, FLNSP4-Ala6, FLNSP4-HydroMut, NSP4_46-175,_ NSP4_Ala6,_ NSP4_HydroMut_, Rev1, Rev2, Rev3, Rev2 116,124,127A, Rev2 116A, Rev1,2 127A, and Rev1 124,127A) in the appropriate pD22 and pD32 vectors by a modified lithium acetate procedure as previously described [25,47]. Briefly, *S. cerevisiae* MaV203 was grown in YPAD overnight at 30°C, diluted to an OD_600_ of 0.5, and incubated at 30°C with shaking to an OD_600_ of 2. The cells were washed with _d_H_2_O, centrifuged, and washed with 1 ml of 100 mM lithium acetate (LiAc), centrifuged and resuspended in 100 mM LiAc. Aliquots were centrifuged and the following solutions were added in order: 240 μL of 50% PEG (3350 mw); 36 μL of 1 M LiAc; 25 μL of salmon sperm DNA (2 mg/ml); 50 μL of _d_H_2_O; and 100 ng of each plasmid DNA. The yeast were shocked at 42°C for 45 min and then incubated at 30°C for 1 h before plating onto complete synthetic medium lacking leucine and tryptophan (CSM Leu^-^Trp^-^).

The transformed yeast colonies were grown at 30°C for 3 days on CSM Leu^-^Trp^-^ to identify colonies containing both plasmids. The activation of transcription of three independent reporter genes (*URA, His3,lacZ)* were monitored by the observation of yeast growth patterns on specific media. *URA3* was monitored by cell growth patterns on CSM Leu^-^Trp^-^Ura^-^ and CSM Leu^-^Trp^-^ + 0.2% 5-fluoroorotic acid (FOA); 5FOA inhibits the growth of yeast. When *HIS3* was transcribed, the growth of the co-transformed yeast was inhibited in a dose-dependent manner by adding 3-amino-1, 2, 4-triazole (3AT) to CSM Leu^-^Trp^-^His^-^[50]. MaV203 expresses a basal level of *HIS3* that was suppressed with increasing concentrations of 3AT to allow discrimination of *HIS3* activation.

Activation of the *lacZ* promoter was detected with a qualitative assay using the substrate X-gal (5-bromo-4-chloro-3-indolyl-β-D-galactopyranoside). To quantitatively measure beta-galactosidase activity, chlorophenol red-beta-D-galactopyranoside (CPRG) was used for the substrate as described in the ProQuest™ Two Hybrid System manual [25,47].

### Expression of FLNSP4, NSP4_46-175_ and a panel of NSP4 mutants

The entry vectors encoding FLNSP4, FLNSP4-Ala6, FLNSP4-HydroMut, and NSP4_46-175_ were used to shuttle the NSP4 sequences into the inducible yeast expression plasmid, pYES-DEST52 (Invitrogen) and transformed into the inducible yeast strain, InVSc-1 [25]. Since our previous data demonstrate stronger binding of NSP4 to cav-1 in Y2H assays when the first two NSP4 N-terminal hydrophobic domains are deleted, we continued our experiments with the truncated NSP4 [25] and constructed NSP4_Ala6_ and NSP4_HydroMut_ for use in the Y2H experiments. All additional mutants (Rev1, Rev2, Rev3, Rev2 116,124,127A, Rev2 116A, Rev1,2 127A, and Rev1 124,127A) were constructed using site directed mutagenesis of NSP4_46-175_, introduced into the Gateway® entry vector, and cloned into the inducible yeast expression plasmid, pYES-DEST52, as above (Table 1). The transformed yeast colonies were grown on CSM Ura^-^ and the encoded NSP4 proteins were induced with YPAG medium **y**east extract, **p**eptone (Difco), 0.01% **a**denine sulfate and 2% **g**alactose (Sigma). Cells were grown in YPAD at 30°C for 24 h, washed and re-suspended to an OD_600_ of 0.5 in YPAG and incubated at 30°C for 24 h. Yeast lysates were prepared using the Zymo Yeast Protein Extraction kit (Zymo Research, Orange, CA) as previously described and were utilized in the binding assays [25]. Approximately 1 × 10^6^ cells were pelleted, Y-Lysis buffer and zymolase were added to the samples and incubated at 37°C for 1 h. The yeast were centrifuged at 400×*g* for 5 min, resuspended in PBS (pH 7.2), containing protease inhibitors 100 μM AEBSF, 80 μM aprotinin, 5 μM bestatin, 1.5 μM E-64, 2 μM leupeptin, 1 μM pepstatin A and 100 μM PMSF (Calbiochem-Novabiochem Corp., San Diego, CA)] and quantified by BCA (Pierce). These lysates were used in the *in vitro* peptide binding and Western blot assays as described below.

### Peptide synthesis, purification and characterization

All peptides were synthesized by fluorenylmethoxycarbonyl (Fmoc) solid-phase chemistry with either 1-hydroxy-benzotriazole (HOBt), O-Benzotriazole-N,N,N’,N’-tetramethyluronium-hexafluoro-phosphate (HBTU) and N,N-diisopropylethylamine (DIPEA), or HOBt and N,N’-diisopropylcarbodiimide (DIPCIDI) activation using the Model 90 Peptide Synthesizer (Advanced Chemtech/AAPTEC; Louisville, KY) as previously reported [32]. Following synthesis, the peptides were cleaved from the solid resin support and all side-chain protecting groups removed by addition of Reagent R (90% trifluoroacetic acid (TFA), 5% thioanisole, 3% ethanedithiol and 2% anisole). Peptides were separated from the solid support by filtration into cold diethyl ether in a dry ice/ethanol bath. Following three organic precipitations, peptides were purified from organic contaminants and incomplete peptide fragments by gravimetric gel filtration chromatography (Sephadex G25 medium) and reverse-phase HPLC using a reverse phase C4 Delta Pak (Waters Chromatography Division, Milford, MA) or C18 (Beckman-Coulter, Fullerton, CA) column. Peptide characterization was by matrix-assisted laser desorption/ionization (MALDI) mass spectrometry (Laboratory for Biological Mass Spectrometry, Department of Chemistry, Texas A&M University, College Station, TX). Mass chromatograms revealed if the full-length peptide was present, the mass of the peptide and the extent of contaminants in the eluted fraction.

All purified peptide samples were tested for endotoxin by the *Limulus* Amebocyte Lysate (LAL) test (Associates of Cape Cod, Inc.) according to the manufacturer’s protocol, to ensure that the samples were endotoxin free. A value of 0.5 EU/mL or less was considered acceptable for use in the diarrhea studies.

### Diarrhea induction in mouse pups

To examine the biological relevance (enterotoxic activity) of the mutations made in the hydrophobic face of the amphipathic alpha-helix/enterotoxic peptide/cav-1 binding domain of NSP4, the NSP4_HydroMut112-140,_ NSP4_AlaAcidic112-140_, NSP4_AlaBasic112-140_ and the wt-NSP4_112-140_ peptides were tested for diarrhea induction as previously described (5, 54) (Additional file 1: Table S1). Briefly, each of the NSP4-specific peptides (100 nmol in 50 μl total volume) was administered by intraperitoneal (IP) delivery to 6-10 day old mouse pups (5-6 pups per peptide). Mouse pups injected with endotoxin-free PBS served as a negative control. Mouse pups were monitored for diarrhea every 2 hours for 12 hours and then at 24 hours post injection. The severity of the diarrhea was scored on a scale of 1 to 4 as previously described (5).

### Western blot assays

The co-transformed yeast colonies were grown in liquid CSM Leu^-^ Trp^-^, and yeast protein extracts were prepared using the Zymo Yeast Protein Extraction kit (Zymo Research, CA). The cell pellets were resuspended in PBS (pH 7.2) containing protease inhibitors and quantified by BCA (Pierce). All lysates were separated by 12% SDS-PAGE, electroblotted onto nitrocellulose membranes, probed with NSP4 or cav-1 peptide-specific antibodies, and reactive bands visualized by the addition of HRP-conjugated IgG and Super Signal® West Pico chemiluminescent substrate (Pierce) followed by exposure to Kodak X-OMAT film [51,52].

### Alignment of NSP4 amino acid sequences

To determine which amino acids in NSP4 residues 113-135 were conserved in different viral strains that encompass RV groups A through E, an alignment was generated of RV sequences obtained from [GenBank: ABZ04174, BAA24144, ABZ04170, BAB83830, AAA64924, AAL11029, AAB58698, AAD50676, ABV66094, BAA13728, P08434, AND P04512]. The sequences were aligned using SDSC Biology Workbench (http://workbench.sdsc.edu) with CLUSTALW for multiple sequence alignments [51]. The percent identities with respect to SA11 were deduced using MView 1.4 [53].

## Discussion

This study demonstrated that the hydrophobic face of the AAH of the enterotoxic domain of NSP4 is critical for the binding to cav-1 and structure may play a role. An NSP4 construct that disrupted the charged face of the amphipathic helix (NSP4-Ala6) continued to bind cav-1, but alteration of three residues (I113R, V124K, Y131D) in the hydrophobic face disrupted cav-1 binding (NSP4-HydroMut). When any one of the three mutated hydrophobic amino acids was restored, NSP4 binding to cav-1 was restored as observed with the Y2H and peptide binding assays. We had not anticipated that all three hydrophobic amino acids selected for mutation in NSP4-HydroMut would individually restore the binding to cav-1, but anticipated reversion of more than one of the three mutated residues would be required to reconstitute the binding site. All three revertants included L116 and L127 suggesting these hydrophobic residues were critical to cav-1 binding. Further analysis of L116 and L127, using the revertant mutants that substituted L116A and/or L127A identified L116 as a key residue for NSP4 binding to cav-1. The ability of L116 to bind cav-1 changes the reactivity from binding in native NSP4 to not binding in NSP4_HydroMut_, to binding in the Rev 2 and finally to not binding in Rev2 with mutated L116A (Figure 1C).

Yet, NSP4_HydroMut_ contained L116 and failed to bind cav-1, suggesting more than one amino acid is required to accomplish binding. Protein-protein interactions can rely on the recognition of linear motifs yet, binding sites frequently depend on (a) exposure of the binding motif at the surface of the protein; (b) the environment of the binding residues; and (c) may require conformation-dependent epitopes for binding [54]. Given that the binding site is within the hydrophobic face, typical protein folding patterns would not place these residues on the protein surface, but would protect them from the aqueous environment of the cell. Perhaps the NSP4-cav-1 binding site was dependent on the secondary structure of a region that included L116 that was disrupted in the NSP4_HydroMut_. The CD analysis confirmed that the NSP4_HydroMut_ peptide has a different secondary structure than the NSP4 wild type peptide.

Notably the identified NSP4 residue that binds cav-1 overlaps the enterotoxic peptide. To begin to understand the functional connection between NSP4 enterotoxic activity and cav-1 binding, diarrhea induction in neonatal mice was evaluated with peptides corresponding to the wt-NSP4_112-140_, NSP4_AlaAcidic112-140_, NSP4_HydroMut112-140_ and NSP4_AlaBasic112-140_. The NSP4_AlaBasic112-140_ and NSP4_HydroMut112-140_ peptides failed to induce diarrhea, whereas when three acidic residues were altered to alanines, NSP4_AlaAcidic112-140,_ diarrhea was induced, similar to the wild type peptide. As the CD analysis of NSP4_AlaAcidic112-140_ and NSP4_AlaBasic112-140_ indicated disruption of the AAH, the helix appeared to tolerate changes in the charged face. Computer modeling of the mutants using PyMol showed constraints for structure especially with I113R (data not shown). These data support the model that the integrity of the secondary structure of the hydrophobic face of the AAH of NSP4 may be important for the induction of diarrhea.

Despite our diarrhea data, it is unclear if cav-1 binding in itself plays a role in diarrhea induction or if cav-1 directs NSP4 to caveolae for signal induction. A previous study highlighted the importance of the tyrosine residue in the hydrophobic face of the AAH. A synthetic peptide in which the tyrosine residue was mutated to a lysine lacked diarrhea-inducing function [55]. In this study, when tyrosine was changed to aspartic acid (D), NSP4 failed to induce diarrhea. Although Y131 is important for the induction of diarrhea in some but not all strains of RV [55], Y131 appears to be dispensable for cav-1 binding. Both Rev1 and Rev2 bind cav-1 in the absence of Y131, and the lack of Y131 does not influence cav-1 binding of Rev1,2 127A or Rev1 124,127A. Our data herein supports and further suggests that the structure of the AAH of NSP4 may play a significant role in promoting diarrhea.

A recent study revealed that the α1β1 integrins are receptors for NSP4 [12]. Binding to these receptors initiates cellular signaling processes (in particular, activation of phospholipase C) that mobilize calcium and result in diarrhea induction. While NSP4 binds the integrin receptor at residues 114-135, signaling occurs via an interaction between NSP4 131-140 and a separate integrin domain [12]. NSP4 HydroMut_112-140_ contains a mutation at aa131, which is within the integrin signaling domain, and does not cause diarrhea. Lack of enterotoxic function with this mutant peptide may be a result of its inability to initiate integrin mediated signaling. These results agree with previous data demonstrating that the exogenous addition of NSP4 mobilizes [Ca^2++^]_I_ from ER stores through a phospholipase C-inositol 1,4,5-triphosphate (PLC-IP_3_) pathway [6].

The data presented suggested that the integrity of the hydrophobic face of NSP4 was critical to both cav-1 binding and modulating diarrhea induction. Localization of the binding site to the conserved hydrophobic residues between RV strains and the lack of diarrhea induced by the synthesized peptide with the corresponding mutations in the hydrophobic face of NSP4 indicated the importance of the cav-1 binding site of NSP4. However, the lack of diarrhea observed with the peptide NSP4_AlaBasic112-140_ indicated that the NSP4 enterotoxin might extend into the basic face. Additional studies are needed to dissect the complexity of NSP4’s multiple activities and binding domains, and how these domains are coordinated during a viral infection.

### Ethics

Research was conducted in compliance with the Animal Welfare Act and other federal statutes. The authors adhered to the principles stated in the *Guide for the Care and Use of Laboratory Animals,* National Research Council. Animals were housed in a fully accredited facility.

## Abbreviations

AAH: Amphipathic alpha helix; AD: Activating domain; BD: Binding domain; 5-FOA: 5-Fluoroorotic Acid; 3AT: 3-Amino-1,2,4-triazole; RV: Rotavirus; NSP4: Nonstructural protein 4; Hobt: O-Benzotriazole-N,N,N’,N’-tetramethyluronium-hexafluoro-phosphate; Fmoc: Fluorenylmethoxycarbonyl; HBTU: 1-hydroxy-benzotriazole; DIPEA: N,N-diisopropylethylamine; DIPCIDI: N,N’-diisopropylcarbodiimide.

## Competing interests

The authors declare the absence of competing interests.

## Authors’ contributions

RDP and CVW conducted the Y2H and associated assays (cloning, peptide pull-downs, etc.). MES and JMB prepared the synthetic peptides and peptide-specific antibodies, and completed CD analyses. Computer alignments were completed by RDP. JMB and RDP were the primary authors of the manuscript and JMB conducted the diarrhea study. RDP prepared the PyMol structures. All authors read and approved the final manuscript.

## Supplementary Material

Additional file 1: Table S1 Percent α-helix of wtNSP4 112-140 and mtNSP4 112-140 peptides in aqueous buffer, 50% TFE and diarrhea induction. Click here for file
