# Supplementary material for: Mutational analysis of the rotavirus NSP4 enterotoxic domain that binds to caveolin-1
Source: Virol J. 2013 Nov 13;10:336. doi: 10.1186/1743-422X-10-336 (PMC3924327; doi:10.1186/1743-422X-10-336)
Supplement: Additional file 1: Table S1 — Percent α-helix of wtNSP4 112-140 and mtNSP4 112-140 peptides in aqueous buffer, 50% TFE and diarrhea induction. [file 1743-422X-10-336-S1.pdf]

Additionalfile1:Table S1 Percent α-helix of wtNSP4 112-140 and mtNSP4 112-140 peptides in aqueous buffer, 50% TFE and [illegible] induction

Peptide:	Peptide Sequence (N to C)[a]	Aqueous Buffer[b]	50% TFE[c]	% Diarrhea induction[d]
$wtNSP4_{112-140}$	MIDKLTTREIEQVELLKRIYDKLTVQTTG	30.6 ± 2.4%	79.6 ± 4.6%	50 (3/6)
$NSP4_{HydroMut112-140}$	M***R***DKLTTREIEQ***K***ELLKRI***D***DKLTVQTTG	19.4 ± 2.2%	57.8 ± 0.6%	0 (0/6)
$NSP4_{AlaAcidic112-140}$	MI***A***KLTTREIEQV***A***LLKRIY***A***KLTVQTTG	23.7 ± 0.6%	73.1 ± 7.3%	0 (0/6)
$NSP4_{AlaBasci112-140}$	MID***A***LTT***A***EIEQVELLKRIYD***A***LTVQTTG	26.2 ± 2.9%	75.3 ± 2.8%	67 (4/6)
Negative control	PBS only, no peptide			0 (0/4)

a. Amino acid residues of the mutated $NSP4_{112-140}$ peptides are listed. The residues that are red in color and bold italics replaced the corresponding amino acids in the wt $NSP4_{112-140}$ peptide. The residues that are blue in color and bold italics replaced the corresponding amino acids in the wt $NSP4_{112-140}$ peptide.
b. b. The peptides were solubilized in an aqueous buffer, phosphate buffered saline (PBS), and individually tested for percent alpha-helix structure as determined by circular dichroism. Data are presented as the mean % alpha-helical content ± SD, n=4.
c. c. The peptides were solubilized in 50% trifluoroethanol (TFE) buffer and individually tested for percent alpha-helix structure as determined by circular dichroism. Data are presented as the mean % alpha-helical content ± SD, n=4.
d. d. The peptides were solubilized in PBS, injected into mouse pups (6-10 day old) using the intraperitoneal route, and observed for the induction of diarrhea. Data are presented as per cent diarrhea produced in four to six mouse pups tested per peptide.
